# Inpatient GHB withdrawal management in an inner-city hospital in Sydney, Australia: a retrospective medical record review

**DOI:** 10.1007/s00213-022-06283-6

**Published:** 2022-12-12

**Authors:** Krista J. Siefried, Georgia Freeman, Darren M. Roberts, Rhiannon Lindsey, Craig Rodgers, Nadine Ezard, Jonathan Brett

**Affiliations:** 1 The National Centre for Clinical Research On Emerging Drugs (NCCRED), The University of New South Wales, Randwick Campus 22/32 King St Randwick, Sydney, NSW 2031 Australia; 2 The National Drug and Alcohol Research Centre (NDARC), The University of New South Wales, Sydney, Australia; 3 Alcohol and Drug Service, St Vincent’s Hospital Sydney, Darlinghurst, Australia; 4 Department of Clinical Pharmacology and Toxicology, St Vincent’s Hospital Sydney, Darlinghurst, Australia; 5 St Vincent’s Clinical School, The University of New South Wales, Sydney, Australia; 6 Drug Health, Royal Prince Alfred Hospital, Sydney, Australia; 7 Faculty of Medicine, Notre Dame University, Sydney, Australia; 8 New South Wales Drug and Alcohol Clinical Research and Improvement Network (DACRIN), Sydney, NSW Australia

**Keywords:** Gamma-hydroxybutyrate, GHB, Gamma-butyrolactone, GBL, 1,4-butanediol, 1,4BD, Withdrawal, Pharmacotherapy, Oxybate

## Abstract

**Rationale:**

Regular consumption of gamma-hydroxybutyrate (GHB) may result in a dependence syndrome that can lead to withdrawal symptoms. There are limited data on medications to manage GHB withdrawal.

**Objectives:**

To examine characteristics associated with delirium and discharge against medical advice (DAMA), in the context of implementing a GHB withdrawal management protocol at an inner-city hospital in 2020.

**Methods:**

We retrospectively reviewed records (01 January 2017–31 March 2021), and included admissions that were ≥ 18 years of age, admitted for GHB withdrawal, and with documented recent GHB use. Admissions were assessed for demographics, medications administered, features of delirium, ICU admission, and DAMA. Exploratory analyses were conducted to examine factors associated (*p* < 0.2) with features of delirium and DAMA.

**Results:**

We identified 135 admissions amongst 91 patients. Medications administered included diazepam (133 admissions, 98.5%), antipsychotics (olanzapine [70 admissions, 51.9%]), baclofen (114 admissions, 84%), and phenobarbital (8 admissions, 5.9%). Features of delirium were diagnosed in 21 (16%) admissions. Delirium was associated with higher daily GHB consumption prior to admission, while duration of GHB use, time from presentation to first dose of diazepam, and concomitant methamphetamine use were inversely associated with delirium. DAMA occurred amongst 41 (30%) admissions, and was associated with a longer time from presentation to first dose of baclofen, while being female and receiving a loading dose of diazepam were inversely associated.

**Conclusions:**

This study adds to the literature in support of the safety and feasibility of diazepam and baclofen for the management of GHB withdrawal. Prospective, randomised trials are required.

## Introduction

A naturally occurring neurotransmitter, gamma-hydroxybutyrate (GHB), is both a precursor and metabolite of GABA (Schep et al. 2012). It can cause desired effects such as euphoria and disinhibition but, at higher doses, GHB becomes a potent central nervous system (CNS) depressant (Schep et al. 2012). GHB (and its precursors gamma-butyrolactone [GBL] and 1,4-butanediol [1,4-BD]) is an illicit substance used for many reasons, and often in combination with other drugs, including as a “party drug”, to facilitate sex-based sociality (known as “chemsex” or “party and play”) (Bourne et al. 2014), and for self-management of sleep and anxiety. Medically, the sodium salt of GHB, sodium oxybate, is used in the USA and some European countries for the treatment of narcolepsy, and in some countries for the treatment of alcohol use disorder and withdrawal (Fuller and Hornfeldt 2003; Wedin et al. 2006). In the most recent Australian population household survey (2019), approximately 1% of respondents 14 years and older reported any lifetime use of GHB (Australian Institute of Health and Welfare 2020). However, amongst convenience samples of some demographic groups, such as lesbian, gay, and bisexual people, rates 20–54 times greater have been recorded (Hammoud et al. 2018; Mooney-Somers et al. 2020). A global systematic review identified growing evidence for GHB-related adverse effects amongst people who use GHB regularly (Dijkstra et al. 2021). Harms associated with the use of GHB include overdose and use disorder (Schep et al. 2012). Harms are increasing worldwide (United Nations Office on Drugs and Crime 2018). This includes in Australia, where ambulance call-outs (Arunogiri et al. 2020) and emergency department presentations for GHB overdose (Harris et al. 2021) have risen over the past decade, and a national priority setting study identified GHB overdose and withdrawal as a key clinical research priority in the alcohol and other drug sectors (Siefried et al. 2021).

People who consume GHB regularly may experience a dependence syndrome that can result in typical withdrawal symptoms (Kamal et al. 2017). The onset of withdrawal can be rapid—commencing within an hour of discontinuation and peaking at 24 h—and can last up to 3 weeks (Schep et al. 2012). While the clinical characteristics of a withdrawal syndrome have not been well defined in the literature, case studies and reviews describe similarities between withdrawal symptoms of GHB and other CNS depressants such as alcohol or benzodiazepines (Wojtowicz et al. 2008; Wolf et al. 2021). The Diagnostic and Statistical Manual of Mental Disorders, Fifth Edition (DSM-5), characterises a withdrawal syndrome associated with sedative, hypnotic, or anxiolytic drugs by two or more symptoms including autonomic hyperactivity (e.g. increased temperature, heart rate, respiratory rate, blood pressure), tremor, insomnia, nausea, anxiety, and psychomotor agitation (American Psychiatric Association 2013). Other symptoms include audio/visual/tactile hallucinations, paranoid delusions, and seizures (American Psychiatric Association 2013; Kamal et al. 2017). Renal failure (Bhattacharya et al. 2011), cardiac arrest, and death (Dyer et al. 2001) have all been reported in GHB withdrawal (Wojtowicz et al. 2008).

Due to the potential for severe physical or psychiatric complications, inpatient medical management of GHB withdrawal may be warranted for people who regularly consume high amounts (van Noorden et al. 2015), although outpatient withdrawal management has been documented. A number of possible risk factors for GHB withdrawal complications have been identified, such as frequent intervals of GHB dosing prior to cessation (Kamal et al. 2017); concomitant psychostimulant use (Kamal et al. 2016a); or female gender (Wolf et al. 2021). However, most studies report infrequent rates of delirium and are underpowered to examine for associated factors (Dijkstra et al. 2017; Kamal et al. 2016a), particularly studies examining tapering GHB pharmacotherapy, which likely ameliorates symptoms of withdrawal such as delirium (Wolf et al. 2021).

In the absence of randomised clinical trials, the existing literature reports on case studies and open-label treatment protocols. Pharmacological interventions for the management of GHB withdrawal are guided by the mechanism of action of GHB on the GABA system, including high-dose benzodiazepines (see narrative reviews by Busardò and Jones (2015); Kamal et al. (2016b); Kamal et al. (2017); Schep et al. (2012); Wood et al. (2011)); titration and tapering with pharmaceutical GHB (sodium oxybate) (not available for use in Australia) (Dijkstra et al. 2017; Kamal et al. 2017); barbiturates such as phenobarbital (particularly if symptoms are resistant to benzodiazepines; reported in limited case reports) (Ghio et al. 2014); or the GABA-B receptor agonist baclofen (reported in case series) (LeTourneau et al. 2008). Less commonly discussed are gabapentin and antipsychotics (Kamal et al. 2016b).

Given the lack of large-scale randomised trials, withdrawal scales, or treatment protocols, the management of GHB withdrawal can vary, although there is a pharmacological rationale for the aforementioned regimens. We sought to review the clinical experience at an inner-urban hospital, where in 2020 a procedure for the management of GHB withdrawal was introduced in an attempt to standardise practice. The procedure includes benzodiazepines, baclofen, and phenobarbital, as outlined below. The present study aims to determine the outcomes of patients presenting with GHB withdrawal including features of delirium and discharge against medical advice (DAMA) prior to and following implementation of the procedure, and the characteristics associated with delirium and DAMA.

## Methods

### GHB withdrawal procedure

The inpatient GHB withdrawal procedure commences with a clinical review by a Registered Nurse and Addiction Medicine Specialist. This includes assessment of medical history and comorbidities, and current drug and alcohol use including other CNS depressants (e.g. alcohol, benzodiazepines). Where indicated based on a prediction of risk of withdrawal severity, planned admission to the inpatient withdrawal unit is facilitated. This is based on dosing pattern, duration of continuous use and volume of GHB consumption, history of complicated GHB withdrawal, or other medical or psychiatric comorbidities. Patients can remain as inpatients in the specialist withdrawal treatment unit for 7 days, or longer if medically indicated. The procedure includes a loading dose of oral diazepam (20 mg every 1–2 h to a recommended 60 mg or until lightly sedated), commenced as early as possible relative to the last GHB dose and ideally prior to the onset of withdrawal symptoms. This is followed by a maintenance dose of diazepam 10–20 mg hourly to a maximum of 120 mg in a 24-h period (tapering over the admission). Baclofen (10–25 mg three times daily) can be commenced concurrently with benzodiazepines when a moderate or severe withdrawal is expected. If a patient requires more than 200 mg of diazepam in a 24-h period, this prompts an intensive care review and consideration of barbiturate therapy. An oral loading dose of 30 mg phenobarbital hourly (maximum 120 mg) until lightly sedated then 30 mg two hourly (max 240 mg) is given to patients in the withdrawal unit or general medical ward. Intravenous phenobarbital is recommended for patients admitted to intensive care with boluses of 5-10 mg/kg two hourly until sedated. Once stabilised on baclofen, patients are weaned from benzodiazepines by tapering the dose over 7 days. Patients are not discharged on benzodiazepines; these are ceased upon discharge. If continued on discharge, baclofen is weaned by tapering the dose over several weeks at the treating specialist’s discretion.

### Included records

We conducted a retrospective medical record review of patients admitted to an inner-urban tertiary hospital in Sydney, Australia, with GHB withdrawal for the period of 01 January 2017 to 31 March 2021. Patient records were identified by a medical record search for admissions that were coded for withdrawal, which coded GHB as the primary drug of concern. Patient records were included in the study if they met the eligibility criteria: admitted as an inpatient, diagnosed with GHB withdrawal, documented recent GHB use at least two times per week, and at least 18 years of age.

### Variables assessed

Records were assessed for medications administered, features of delirium, ICU admission, discharge against medical advice, and re-presentation to a hospital (limited only to data for the admitting hospital) within the study period. Exploratory analyses were conducted to examine factors associated with features of delirium and DAMA.

Demographic and other data collected from the included medical records were age, sex, gender, sexuality, relationship status, known legal issues, GHB use and history (prior history of GHB use, prior history of GHB overdose, prior history of GHB withdrawal, age of first GHB use, date and time of last GHB use [estimated or actual], GHB use pattern [frequency, duration, dose, nocturnal use]), other substance use (frequency, dose, age first use, date last use, prior withdrawal), intravenous drug use, comorbidities, concomitant medications, date and time of admission, reason for admission, number of admissions (e.g. if prior admissions recorded), pharmacological management of GHB withdrawal (medication given, first [loading] dose amount, maintenance dose amount, time of first dose, total daily dose, discharge prescription, free text for notations), intensive care assessment/admission (binary yes/no—if yes: date and time, discharge date and time, sedation, intubation), delirium diagnosis (binary yes/no—if yes: features, resolved, free text for notations), adverse events (binary yes/no—if yes: details, resolved), code black called for aggressive/self-harming behaviour, discharge status (with a plan, or against medical advice), relapse prevention plan, follow-up post-discharge, last follow-up date, and known GHB use post-discharge.

### Statistics

Included records were manually reviewed and data entered into a RedCap database (Harris et al. 2009). Descriptive statistics were used to describe the sample. Logistic regression analysis was conducted to determine factors associated with the outcomes of delirium, and discharge against medical advice. Given patients could re-present, generalised linear models with generalised estimating equations and logit link functions were used with an exchangeable correlation structure to account for within-patient correlations. Bivariate regression was conducted on all variables and those with a *p*-value ≤ 0.2 were included in a multivariate regression analysis. Variables were removed by backward elimination based on their significance (two-sided alpha *p* < 0.2) (Heinze et al. 2018) and model fit based on the Akaike information criterion (AIC). Given that this is an exploratory analysis, all variables with a *p*-value < 0.2 were retained in the multivariate analysis and are reported, irrespective of 95% confidence intervals. Data were analysed in R version 3.6.2 (2019–12-12).

### Ethics

The St. Vincent’s Hospital Human Research Ethics Committee approved the study (ETH: 02,221) and given the methodology (retrospective record review) a waiver of consent was approved. This report follows the STROBE statement for reporting observational studies (von Elm et al. 2014).

## Results

### Included patient admissions

**Fig. 1:**
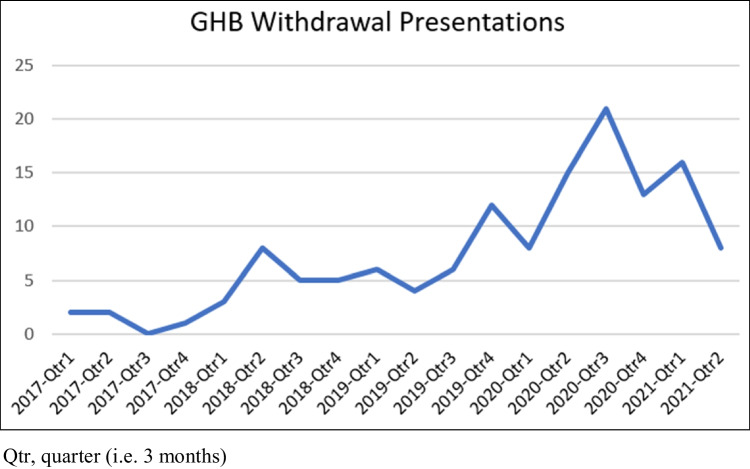
GHB withdrawal presentations over time

One-hundred and thirty-five admissions relating to admissions of 91 patients met our eligibility criteria and were included in our review. Forty-two patients (46.2%) were male; the median age at first admission was 31 years (range: 19–53 years). The median age of first GHB use was 27 years (range: 15–46). The median amount of GHB consumed daily in the days prior to admission was 30 mL (range: 9–120 mL). Over half of the admissions involved nocturnal dosing of GHB (*n* = 70 [52%]), and 83 patients (91%) consumed GHB daily. Eighty-seven patients (96%) consumed other substances, most commonly methamphetamine (*n* = 76, 84%) and benzodiazepines (*n* = 36, 40%). Ninety-four (70%) admissions were an elective admission for withdrawal, 41 (30%) were unplanned (i.e. via the emergency department), and seven (5%) of these were related to a GHB overdose. Demographics are detailed in Table 1, and presentations over time are presented in Fig. 1.

**Table 1:** Sample characteristics

	Sample (*n* = 91) ^a^
Demographics	
Age (years, median [IQR])	31 (19–53)
Sex	
Male	42 (46.2)
Female	49 (53.8)
Other	0 (0)
Sexuality	
Heterosexual	12 (13.2)
Homosexual	4 (4.4)
Not recorded	75 (82.4)
Relationship status	
Single	35 (38.5)
Partnered	23 (25.3)
Not recorded	33 (36.3)
GHB use and history	
Age of first GHB use (years, median [IQR])	27 (15–46)
Prior history GHB overdose	
No	1 (1.1)
Yes	39 (42.9)
Not documented	51 (56)
Prior GHB withdrawal at home	
Yes	23 (25.3)
No	11 (12.1)
Not documented	51 (56)
GHB use frequency	
2–4 times per week	3 (3.3)
5–6 times per week	5 (5.5)
Daily	83 (91.2)
GHB use dose (mL, median [IQR])	30 (9–120)
Other substance use	
Methamphetamine	76 (83.5)
MDMA	2 (2.2)
Cocaine	10 (11)
Opioids/opiates	9 (9.9)
Cannabis	15 (16.5)
Benzodiazepines	36 (39.6)
Alcohol	25 (27.5)
Nicotine	80 (87.9)
Intravenous drug use	
Current	34 (37.4)
Previous	15 (16.5)
Never	42 (46.2)
Hospital admission and outcomes	
Delirium	21 (15.6)
Intensive Care Unit admission	3 (2.2)
Code black ^b^	7 (5.2)
Discharge against medical advice	41 (31)
Legal issues	Presentations (*n* = 135) ^c^
Current	45 (33.3)
Previous	22 (16.3)
Never	20 (14.8)
Not documented	48 (35.6)

^a^Recorded at first presentation

^b^Called when staff determined personal threat

^c^Recorded at every admission

### GHB withdrawal management

Medications administered were benzodiazepines (diazepam [133 admissions, 98.5%]), antipsychotics (olanzapine [70 admissions, 51.9%]), and baclofen [114 admissions, 84%]. Loading doses of diazepam were given in 88 (65.2%) admissions. This was more consistent following the implementation of the procedure (43.5% of admissions prior, and 76.4% following). The median time from presentation to the first diazepam dose was 1.8 h (range 0 to 80.5 h). Baclofen was more commonly administered following the implementation of the procedure (71.7% of admissions prior, and 91.0% of admissions following the procedure). The median time from presentation to the first baclofen dose was 3.2 h (range 0 to 56.5 h). Forty-eight percent of admissions were discharged on baclofen (21.7% of admissions prior, and 61.8% following). Twenty-five admissions (18.5%) indicated that other medications were used, including phenobarbital (8 admissions, 5.9%), clonidine (8 admissions, 5.9%), and other medications (17 admissions, 12.6%).

Twelve admissions (9%) received assessment by an intensive care doctor, three admissions (2.2%) were admitted to the intensive care unit, and two (1.5%) were intubated. The median length of stay in the hospital was 3.5 days (range 0.4 to 32.6 days), and there was little change prior to (3.4 days) and following (3.6 days) the procedure’s implementation. Seventy-four (55%) had a documented relapse to GHB use following discharge, 21 (16%) within 7 days of discharge; however, for 41 admissions (30%), these data were unavailable, as these patients did not have any further documented interaction with our hospital.

### Covariates predictive of delirium

Features of delirium were diagnosed in 21 (16%) admissions. Delirium was present in five admissions (11%) prior to the procedure implementation and 16 admissions (18%) following. The median time from admission to the onset of delirium was 10.1 h (0.6 to 43.7 h). Episodes of delirium lasted for a median of 40.5 h (range 13.0 to 398.2 h); 44.8 h pre- versus 35.3 h post-procedure implementation.

Delirium was associated with higher daily GHB consumption prior to admission (odds ratio [OR] 1.02, 95% confidence interval [95%CI] 1.00–1.04, *p* = 0.097), while the duration of GHB use (OR 0.92, 95%CI 0.83–1.01, *p* = 0.085), concomitant methamphetamine use (OR 0.24, 95%CI 0.08–0.73, *p* = 0.013), and time from presentation to the first dose of diazepam (OR 0.80, 95%CI 0.66–0.98, *p* = 0.032) were inversely associated with delirium.

### Covariates predictive of discharge against medical advice

Discharge against medical advice occurred amongst 41 (30%) admissions, while 14 (10%) discharged prematurely but with a treatment plan. In the 41 admissions who were documented as discharged against medical advice, this was associated with time from presentation to the first dose of baclofen (OR 1.04, 95% CI 0.99–1.08, *p* = 0.099), while being female (OR 0.32, 95%CI 0.12–0.82, *p* = 0.018) and receiving a loading dose of diazepam (OR 0.45, 95%CI 0.17–1.16, *p* = 0.097) were inversely associated with discharge against medical advice.

## Discussion

We analysed 135 admissions for GHB withdrawal, assessing treatments provided and outcomes including features of delirium and discharge against medical advice. Our sample was nearly universally administered diazepam, and following implementation of a procedure for withdrawal management, nearly all received baclofen and almost half were discharged home on ongoing baclofen treatment. The procedure for dosing diazepam and baclofen was safe and feasible in this sample; intensive care admission was rare, and delirium uncommon. In an exploratory analysis, delirium was associated proportionally with the amount of GHB consumed, while the duration of GHB use, concomitant methamphetamine use, and earlier diazepam dosing were inversely related. Discharge against medical advice was less likely amongst females and those who received a loading dose of baclofen, while participants who had a longer time from the presentation to the first dose of baclofen were more likely to discharge against medical advice.

Our sample enrolled cases that were often complex in terms of complications and polysubstance use, with high rates of discharge against medical advice and re-presentation to the hospital for relapse to GHB use, or subsequent GHB withdrawal support. This may be due to the inner-urban location of the hospital, where specialist stimulant treatment services are available and may refer for services related to GHB, combined with the availability of a drug and alcohol withdrawal unit led by addiction medicine specialists. The median length of stay in the hospital was 3.5 days; this and the proportion of discharge against medical advice may be related to the inpatient unit being limited in some respects that may influence a patient’s choice to remain admitted. Contextual factors such as tobacco smoking and the use of vaporised nicotine products not being permitted (although another nicotine replacement therapy is offered to all inpatients) or the inpatient unit being a closed unit (no outside visitors permitted) may be factors associated with early discharge, although this study did not collect the qualitative data required to assess this. Studies in other settings, multi-centre studies, and qualitative studies are required to elucidate reasons for short durations of stay in the inpatient withdrawal unit. We also note that discharge against medical advice is being re-interpreted as premature discharge (Ambasta et al. 2020) and greater exploration is required to understand how patients’ needs can be met, and the relationship between premature discharge and treatment outcomes. Cravings for GHB, urge and the desire to consume GHB could all be theorised to contribute to premature discharge, but in the absence of a validated withdrawal scale or further qualitative work with patients, it is unknown the extent to which these contribute to premature discharge. Furthermore, given the long half-life of some of the withdrawal medications provided (e.g. phenobarbital) early discharge and risk of GHB consumption post-discharge warrant further follow-up.

In Australia, GHB consumption is low in household surveys (1%) (Australian Institute of Health and Welfare 2020) but up to 50 times greater in people who identify as LGBTQ (Hammoud et al. 2018; Mooney-Somers et al. 2020). Our study did not collect data on sexuality, as this is not a minimum data standard for collection by public services (Freestone et al. 2022). Thus, we are unable to compare our sample with the underlying population of LGBTQ people who consume GHB, or identify any emerging trends of increased use amongst heterosexual people. More broadly, it makes it difficult to ascertain if the current public health campaigns, predominantly targeted to LGBTQ Australians, require expansion to other populations. Our sample included a slightly higher proportion of females to males, consistent with a case series of 12 inpatient GHB withdrawals in Australia (Cappetta and Murnion 2019), while a 2007 study of people who use GHB (*n* = 115) reported 60% were male (Degenhardt and Dunn 2008) and recent analysis of 74 deaths attributed to GHB identified 70% as being amongst males (Darke et al. 2020). Our sample, being on average female, older, and consuming a median of 30 ml GHB daily, does not necessarily reflect the known population of people who consume GHB in Australia. However, little to no population-level data are available on the prevalence and demographics of those with GHB use disorder. Given ongoing surveillance projects amongst LGBTQ people, particularly gay or bisexual men (Hammoud et al. 2017), further monitoring of other populations and people who are experiencing harms associated with GHB (e.g. withdrawal) is required.

While delirium was identified in less than one in five admissions, other studies have demonstrated similar or lower rates of delirium, reporting delirium in 2–21% of participants (Bell and Collins 2011; Beurmanjer et al. 2020; Wojtowicz et al. 2008). Episodes of delirium increased over our study period, and may have reflected that the protocol encouraged clinicians to examine and document features of delirium, thereby reflecting an increase in milder forms of delirium following the implementation of the protocol. While prior studies have identified risks of complicated withdrawal being concomitant methamphetamine use or female gender, we did not find this in our sample, while we did find earlier provision of diazepam to be inversely associated with delirium. However, given the exploratory analysis methods and small sample size, this should be interpreted cautiously. The GHB dose consumed by our sample was relatively low (30 mL median) and may have contributed to the lower prevalence of delirium in this study. Larger studies, enrolling participants with diverse GHB use patterns, or analysis of larger samples that would allow for disaggregation of participants who consumed higher doses, are warranted. Furthermore, unmeasured factors could result in more severe withdrawal presentations.

While a retrospective study cannot evaluate the efficacy of a procedure for GHB withdrawal, the data presented here demonstrate its feasibility and safety in an inpatient setting with intensive care availability. This report aims to improve treatment by transparent reporting to share learning and improve the evidence base for GHB withdrawal treatment given the paucity of evidence to date. We also aimed to inform the future development of GHB withdrawal procedures. This is particularly important given that anecdotally, the severity of dependence increased over the study period, although this was not possible to measure retrospectively. While sodium oxybate has been more extensively studied than other pharmacotherapies for GHB withdrawal (Tay et al. 2022), and is now commonly used in the Netherlands (Beurmanjer et al. 2020), it is not currently registered in Australia, so we have pragmatically evaluated a procedure implementing pharmacotherapies available in our setting. We present the largest review of baclofen in the context of GHB withdrawal. Baclofen has a longer half-life than GHB and is an agonist at GABA-B receptors, and due to its similarity to GHB has demonstrated effectiveness in open-label studies of GHB relapse prevention (Beurmanjer et al. 2018). Establishing the evidence base for administration concomitantly with high-dose benzodiazepines and in the context of anticipated complicated or uncomplicated GHB withdrawal is an important addition to the literature. However, due to the retrospective nature, our study was unable to actively follow up on admitted cases to assess longer-term outcomes associated with baclofen use, including ongoing engagement in care and at services outside of our institution or psychosocial therapy. Furthermore, our procedure did not include a protocol for the tapering of baclofen for cases discharged on baclofen. This study explored the treatment of withdrawal and the prevalence of delirium as a complication. Further prospective studies are required to develop a protocolised management approach—ideally with a scale to more objectively measure withdrawal signs and symptoms and the associations of the treatment of withdrawal and longer-term treatment outcomes.

Our study has limitations. The design is a retrospective data analysis, and the preference would have been to test the procedure and its components prospectively; however, the lower incidence of delirium would require a larger and resource-intensive multi-centre trial of the procedure. Furthermore, the lack of a validated withdrawal measure, standardised approach, or evidence base would make the design for a randomised trial challenging, due to the lack of a “treatment as usual” comparator group. Given the nature of retrospective data collection, data is sometimes missing or incomplete—for example, ideally, the last GHB dose time to the first benzodiazepine time would be recorded but these data were often not documented, as described. Data regarding outcomes post-discharge are not always reliably captured, for example re-presentation to other hospitals is unknown and abstinence from GHB is unknown. Our study also has strengths, while it was relatively small and underpowered, it is the largest case series of GHB withdrawal, reporting on a large number of GHB withdrawal records and analysing treatment with a protocolised regimen, and medical complications, which significantly adds to the literature that mainly comprises case reports and series. However, these results should be interpreted with caution, as there is likely to be unmeasured confounding, and given the low event rates, regression analyses should be treated as hypothesis-generating.

Future research should examine more diverse datasets, possibly by combining data from multiple centres, and linking administrative data to examine outcomes post-discharge. A tool for quantifying the severity of GHB withdrawal which can guide the dosing of pharmacotherapies is also of particular interest. Furthermore, a resource to predict withdrawal severity could streamline management by determining who is at a higher risk of complications (such as delirium), thereby ideally managed in a hospital setting, as opposed to in an outpatient setting. A prospective study to examine outcomes associated with treatment (for example different rates of dose escalation of pharmacotherapies) would provide a valuable evidence base for optimising the treatment of GHB withdrawal.
